# Myocardial injury and pericarditis after combined left atrial and coronary sinus ablation in Wolff–Parkinson–White syndrome: a case report

**DOI:** 10.1186/s12872-020-01333-3

**Published:** 2020-01-17

**Authors:** Mei-fang Zheng, Zhen Wang, Zheng-yu Bao

**Affiliations:** 1 Department of Cardiology, Northern Jiangsu People’s Hospital, No. 98, Nantong West Road, Yangzhou, 225001 Jiangsu China; 2 Clinical Medical College, Dalian Medical University, Dalian, Liaoning China; 3 Clinical Medical College, Yangzhou University, Yangzhou, Jiangsu China; 4 Department of Cardiology, Yangzhou Friendship Hospital, Yangzhou, Jiangsu China

**Keywords:** Wolff–Parkinson–white syndrome, Catheter ablation, Myocardial injury, Pericarditis, Case report

## Abstract

**Background:**

Radiofrequency catheter ablation is an established procedure with a high success rate for treating Wolff–Parkinson–White (WPW) syndrome. Rare complications post-ablation may nonetheless occur particularly associated with coronary sinus. Identifying and avoiding these complications remains a challenge.

**Case presentation:**

A 66-year-old woman with WPW syndrome was admitted to the hospital due to frequent attacks of paroxysmal tachycardia. During electrophysiological study, an accessory pathway was thought to connect the posterior wall of the left ventricle. The patient underwent Radiofrequency (RF) catheter ablation. The procedure was time-consuming because of combined left atrial and coronary sinus ablation. The total amount of radiofrequency application energy in the coronary sinus was 6800 J. After the operation, widespread concave ST-segment elevation, significantly increased value of serum troponin I and mild pericardial effusion were identified, but the patient did not show any symptoms. Therefore, the patient was suspected to have myocardial injury and pericarditis caused by ablation-related injury. The patient was uneventfully discharged five days after the procedure with a significantly decreased value of troponin I. The reexamined electrocardiogram was normal after three weeks.

**Conclusions:**

To the best of our knowledge, this is the first study to report on myocardial injury and pericarditis after combined left atrial and coronary sinus ablation in WPW syndrome. Our findings underscore the need for detailed mapping and careful ablation with low energy, as well as the merits of identifying myocardial infarction after coronary sinus ablation.

## Background

With better awareness and technological advancements, radiofrequency (RF) catheter ablation has been shown to be practical and effective in treating patients with cardiac arrhythmia, such as Wolff–Parkinson–White (WPW) syndrome, supraventricular tachycardia, ventricular tachycardia, and atrial fibrillation [1]. However, rare complications post-ablation may occur, including coronary artery injury, coronary sinus stenosis, and pericarditis, particularly together with coronary sinus and epicardial ablations [2, 3]. The mechanisms underlying these complications are not completely understood. Some cases of pericarditis complications have been reported after atrial fibrillation ablation [4, 5] and ventricular tachycardia ablation [6], but not WPW syndrome ablation. To the best of our knowledge, this is the first report of myocardial injury and pericarditis after combined left atrial and coronary sinus ablation in WPW syndrome. The study findings underscore the need for detailed mapping and careful ablation with low energy, as well as the merits of identifying myocardial infarction after coronary sinus ablation.

## Case presentation

**Fig. 1:**
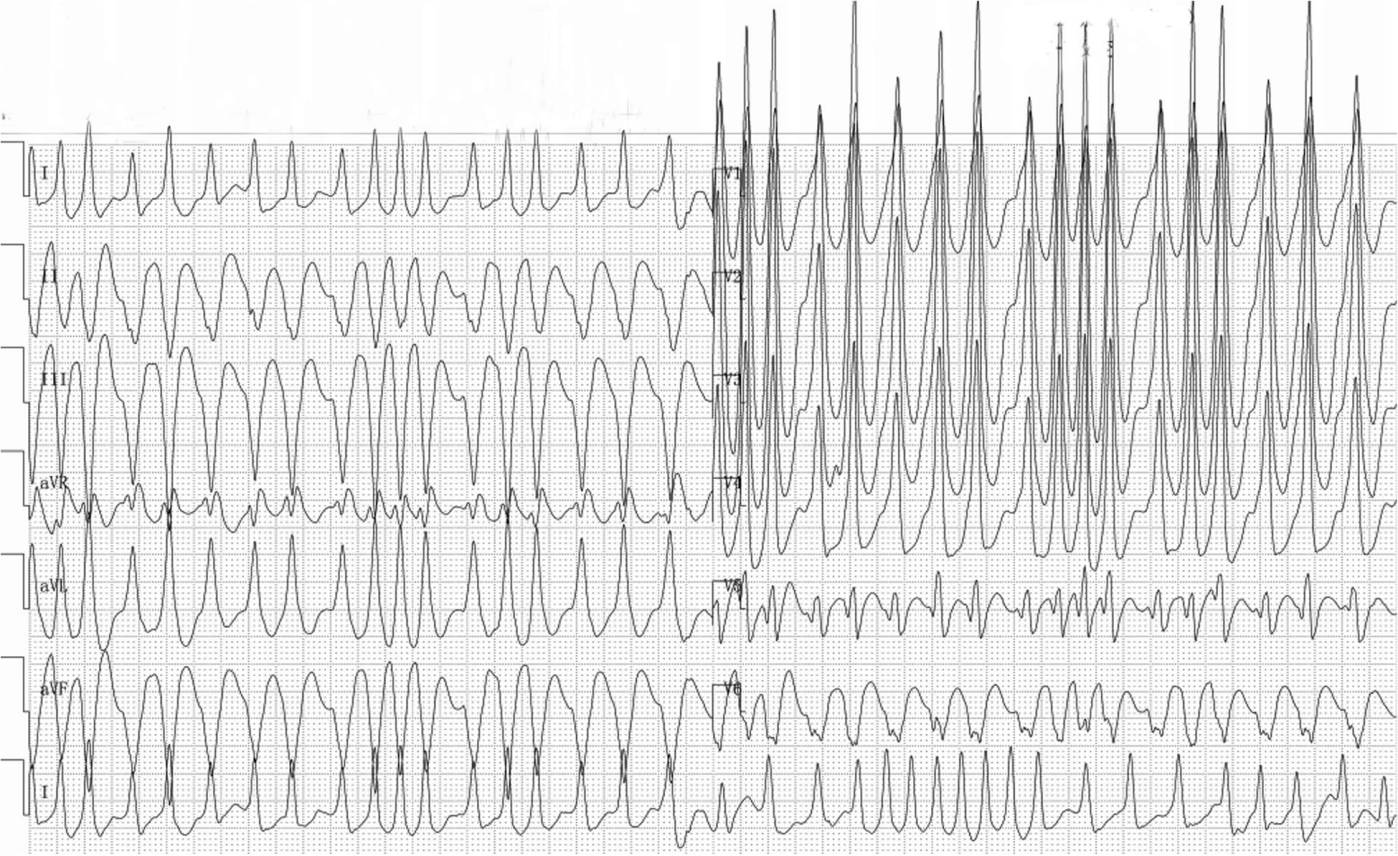
Electrocardiogram showing atrial fibrillation with type A pre-excitation syndrome upon hospital admittance. Delta waves were detected in all leads, and QRS was R-shaped in V1 lead, suggesting left AP

A 66-year-old woman with WPW syndrome was admitted to the hospital because of frequent paroxysmal tachycardia attacks occasionally accompanied by dizziness, which started when she was 58 years old. She suffered repeated chest distress and palpitations for eight years without treatment. An electrocardiogram from the emergency department showed atrial fibrillation with type A pre-excitation syndrome (Fig. 1). The serum troponin I level was 0.415 ng/mL, the creatine kinase isoenzyme level was 5.9 ng/mL, and the myohemoglobin level was 288.3 ng/mL. Physical examination showed a blood pressure of 128/70 mmHg, normal breath sound, normal heart borders, a heart rate of 200 bpm, irregular cardiac rhythm, pulse deficit, and no audible murmur or pericardial friction sound. The patient converted to a sinus rhythm under external direct current cardioversion after unsuccessful drug cardioversion with amiodarone. The echocardiogram after the conversion to sinus rhythm revealed no organic heart disease, an estimated left ventricular ejection fraction (LVEF) of 62%, and mild tricuspid valve regurgitation.

**Fig. 2:**
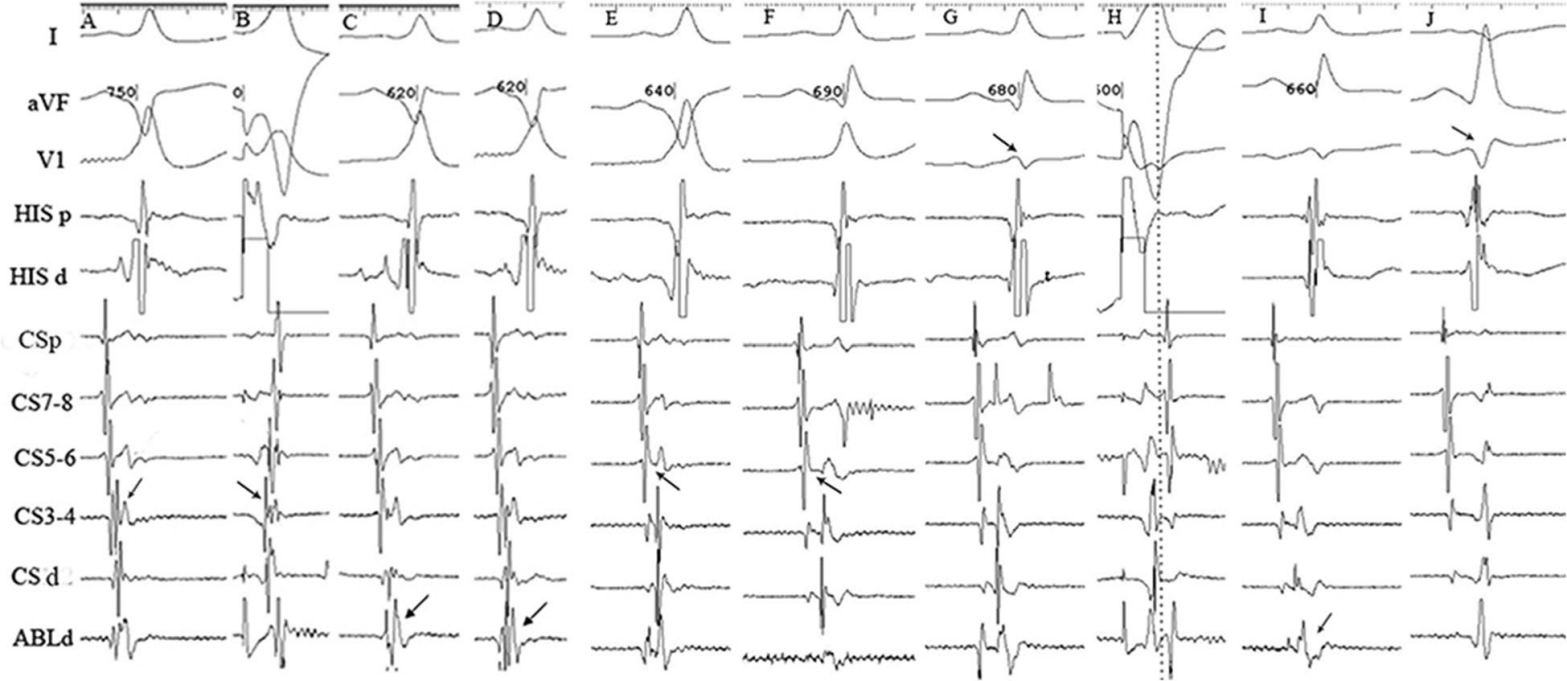
(**A**) The earliest anterograde ventricular activation (V wave) and AP potential in CS34. (**B**) The earliest retrograde atrial activation (A wave) in CS34 during ventricular pacing (RVA S1S1 500 ms). (**C,D**) AV fusion wave in electrogram of ABLd when ablation was performed over left atrial endocardium near CS34 and CS56. (**E,F**) Prolonged AV interval and (**G**) smaller delta wave could be identified during RF applications in CS with RF applications administered near CS34 and CS56. (**H**) Concentric decremental retrograde conduction of V-A waves with RVA S1S1 indicated the block of retrograde conduction of AP. (**I**) AV fusion wave in electrogram of ABLd when ablation catheter was performed over left atrial endocardium near CS34. (**J**) Anterograde conduction of AP disappeared with a completely absent delta wave

Because of the possibility of recurrence of atrial fibrillation with the complication of pre-excitation, the patient underwent RF catheter ablation the following day. A ten-polar coronary sinus (CS) catheter was placed into the CS and an electrophysiological examination catheter was positioned in the right ventricular apex through the left femoral vein. During the sinus rhythm, the earliest anterograde ventricular activation was recorded at the site of CS 34 (the third and fourth poles of the coronary sinus catheter, Fig. 2a). This site was also the earliest retrograde atrial activation site during ventricular pacing (Fig. 2b). Therefore, an accessory pathway (AP) was thought to connect the posterior wall of the left ventricle. The medium curved temperature-controlled bipolar ablation catheter was then inserted into the right femoral vein and advanced to the mitral annulus via the atrial septal puncture. An atrioventricular (AV) fusion wave was identified in the electrogram of the distal end of the ablation catheter (ABLd) when ablation was performed over the left atrial endocardium near CS34 and CS56 (Fig. 2c,d). However, AP conduction remained present after multiple RF applications with power settings ranging between 30 and 40 W. The bipolar ablation catheter was then pulled into the CS and RF applications were administered near CS34 and CS56. Before ablation within the CS, angiography was performed to estimate the CS size. The diameter of the CS proximal end was 7.7 mm while the middle portion was 6.1 mm. With a power setting of 20 W, a prolonged AV interval (Fig. 2e,f) and smaller delta wave (Fig. 2g) were identified in the local electrograms. Concentric decremental retrograde conduction of the V-A wave phenomenon was observed via repeated right ventricular apex extra-stimuli with overdrive pacing mode (RVA S1S1, Fig. 2h). This finding indicated that retrograde conduction of the AP was blocked. Anterograde conduction of the AP could not be completely blocked even after multiple irrigated ablations, because the delta wave did not completely disappear. The total amount of RF application energy in the CS was 6800 J. When the bipolar ablation catheter was again sent to the mitral annulus, an AV fusion wave was identified in the electrogram of ABLd when the catheter was at the 5 o’clock position in the left anterior oblique position of the mitral annulus near CS34 (Fig. 2i). The anterograde conduction of the AP disappeared 4 s later with an RF application power setting of 30 W (Fig. 2j). Anterograde and retrograde conduction of the AP could not be induced by the high-frequency atrial and ventricular stimuli. The patient was free of palpitation attacks, and the electrocardiogram was normal immediately after ablation (Fig. 2j).

**Fig. 3:**
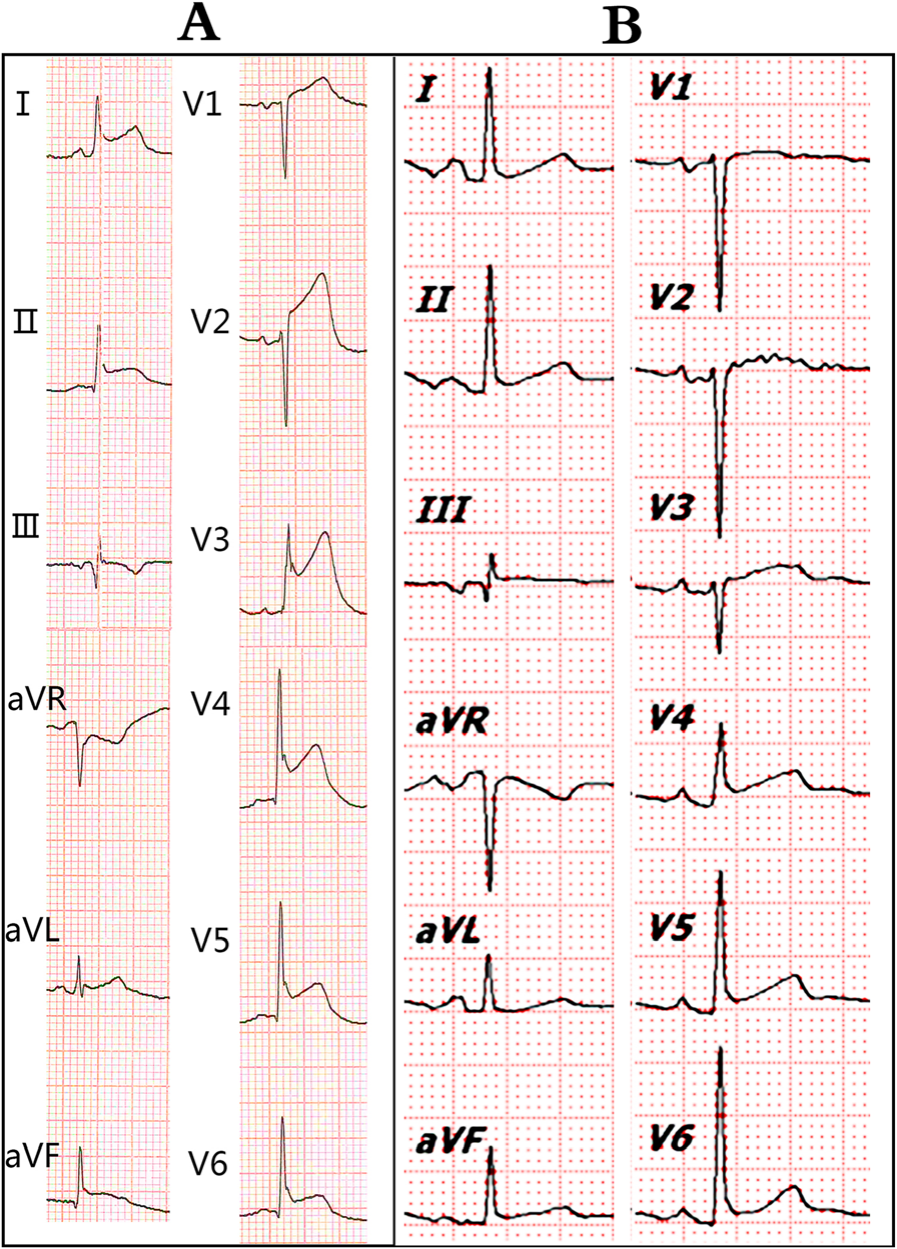
(**A**) Widespread concave ST-segment elevation was present in electrocardiogram after procedure. Except for leads aVR and V1, PR-segment was depressed. (**B**) Normal electrocardiogram three weeks after RF procedure

Another electrocardiogram was performed the following day, revealing a sinus rhythm with frequent atrial premature beats, paroxysmal atrial tachycardia, widespread concave ST-segment elevation, T-wave changes, and left ventricular high voltage (Fig. 3a). The serum troponin I level was 8.700 ng/mL, the creatine kinase isoenzyme level was 23.2 ng/mL, and the myohemoglobin level was 325.0 ng/mL. Repeated electrocardiograms showed sustained widespread concave ST-segment elevation without dynamic changes in QRS and with ST-T morphology. Echocardiograms revealed mild pericardial effusion with an estimated LVEF of 59% and mild tricuspid valve regurgitation. The patient did not show any symptoms such as chest pain or chest distress, thus leading to a suspicion of myocardial injury and pericarditis caused by ablation-related injury. The patient was not scheduled for any specific treatment for pericarditis but was instead monitored, asked to rest and take aspirin 100 mg orally daily for a month which was used to prevent postoperative embolic events. The patient was discharged five days after the procedure with significantly decreased levels of troponin I (0.036 ng/mL) and myohemoglobin (94.7 ng/mL). A repeated electrocardiogram after three weeks revealed normal findngs (Fig. 3b).

## Discussion and conclusions

Myocardial injury and pericarditis are uncommon complications of ablations [4], because heat from the procedure leads to necrosis of the local myocardial cells. In addition, extensive ablation and long-term ablation with high energy may increase the incidence of myocardial injury. Moreover, owing to a thin coronary sinus and surrounding atrium, complications such as myocardial injury, pericarditis, acute myocardial infarction (AMI), and vascular rupture are more likely to occur. In this report, the patient received multiple RF applications and large amounts of RF ablation energy, with ablation areas including the endocardial-coronary sinus. These procedures may have caused myocardial injury and pericarditis in this patient, as characterized by elevated serum levels of myocardial injury markers and an electrocardiogram showing pericarditis. Therefore, ablation should be performed very carefully. To avoid myocardial injury, pericarditis, coronary sinus perforation, and other complications caused by ablation, low RF ablation energy should be applied and the RF applications should be stopped immediately when patients experience chest pain or any other discomfort. If possible, detailed mapping should be performed to maximize the chances of successful ablation with a single burn in WPW.

The possibility of a coronary artery injury must be excluded if there is an elevation in the ST-segment of the electrocardiogram or serum troponin I level is significantly increased after the ablation procedure. Coronary artery injury is a rare complication with a reported incidence of 0.09% among all ablations. Its underlying mechanisms are not completely understood. Several hypotheses have been proposed by experts. One theory suggests that artery damage is inversely correlated with vessel size [7]. Garabelli et al. have proposed that vessels less than 3 mm in diameter are more vulnerable to RF heat, owing to a lack of protection from the heat-sink effect. Certain procedural situations, such as linear ablation within the CS, may also increase this risk [2]. Acute myocardial infarction has been attributed to the close proximity of RF lesions to the coronary artery, especially in the posteroseptal region and CS, as well as its branches. Stavrakis et al. have found an inverse correlation between the risk of coronary artery injury and the distance between the CS ablation site and coronary artery. This injury might be caused by CS muscle conduction [8]. Therefore, care should be taken to avoid ablation within 5 mm of a major coronary vessel. Electrocardiograms, myocardial injury markers, and echocardiography should be routinely reexamined. When elevation in the ST-segment is sudden and the patient experiences persistent chest pain, coronary angiography is indicated to identify complications associated with coronary artery injury. AMI requires immediate percutaneous coronary intervention.

In this case, the patient underwent combined left atrial and coronary sinus ablation and showed higher serum levels of myocardial injury markers and elevated ST-segment in the electrocardiogram post-operation. Identifying whether an AMI occurred and determining a treatment strategy are essential. The electrocardiogram immediately after ablation was normal, and the patient was free of palpitation attacks. Sustained widespread concave ST-segment elevation, rather than convex ST-segment elevation and reciprocal ST-segment depression, was observed. The patient’s electrocardiogram three weeks later was normal without inverted T-waves. The inverted T-wave in AMI typically remains for several weeks to months. Furthermore, a post-operation echocardiogram revealed no organic heart disease but only mild pericardial effusion. Hence, an absence of inverted T-waves, symptoms of chest pain or chest distress, and regional ventricular wall motion abnormalities, as well as the presence of sustained widespread concave ST-segment and pericardial effusion suggested pericarditis rather than AMI. Considering of the absence of chest pain and the self-limiting nature of this kind of pericarditis, the patient was not treated with colchicine or adequate non-steroidal anti-inflammatory drugs (eg. aspirin 750–1000 mg every 8 h) for pericarditis after operation. This patient was scheduled for monitoring and asked to take aspirin 100 mg daily to prevent postoperative embolic events. Finally, she was discharged with no symptom and the reexamined electrocardiogram was normal after three weeks.

In summary, this is the first study to report on myocardial injury and pericarditis after combined left atrial and coronary sinus ablation in WPW syndrome. The findings underscore the need for careful ablation with low energy, especially when combined with CS ablation. It is essential to identify whether an AMI occurred and determining a treatment strategy after coronary sinus ablation. For postoperative patients, the changes in abnormal electrocardiogram and myocardial injury markers should be dynamically monitored together with symptoms and coronary artery angiography should be performed when necessary.

## Data Availability

All the data supporting our findings is contained within the manuscript.

## Abbreviations

ABLd: The distal end of the ablation catheter
AMI: Acute myocardial infarction
AP: Accessory pathway
AV: Atrioventricular
CS: Coronary sinus
LVEF: Left ventricular ejection fraction
RF: Radiofrequency
RVA: Right ventricular apex
WPW: Wolff–Parkinson–white
